# The diagnostic accuracy of wearable digital technology in detecting fertility window and menstrual cycles: a systematic review and Bayesian network meta-analysis

**DOI:** 10.1038/s41746-025-02320-8

**Published:** 2026-01-24

**Authors:** Yue Shi, Chi Chiu Wang, Yongkang Yang, Qin Li, Pui Wah Chung, Yao Wang

**Affiliations:** 1 Department of Obstetrics and Gynaecology, Faculty of Medicine, The Chinese University of Hong Kong, Hong Kong SAR, China; 2 Li Ka Shing Institute of Health Sciences, Chinese University of Hong Kong, Hong Kong SAR, China; 3 Chinese University of Hong Kong-Sichuan University Joint Laboratory for Reproductive Medicine, Hong Kong SAR, China; 4 Second Clinical Medical College, Shaanxi University of Traditional Chinese Medicine, Xianyang, China; 5 Department of Obstetrics and Gynaecology, The Second Affiliated Hospital of Shaanxi University of Traditional Chinese Medicine, Xianyang, China

**Keywords:** Computational biology and bioinformatics, Diseases, Health care, Medical research

## Abstract

This systematic review and Bayesian network meta-analysis assessed the diagnostic accuracy of wearable digital technology (WDT) in monitoring women’s fertility window compared to conventional methods. 8 databases were searched until January 1, 2025. 27 studies were included in the analysis, where 13 studies applied WDT in tracking ovulation. We evaluated the accuracy, sensitivity, specificity, positive likelihood ratio (PLR), negative likelihood ratio (NLR), diagnostic odds ratio (DOR), and summary receiver operating characteristic (SROC) of WDT, and compared the performance of different designs of WDT by NMA analysis. The revised QUADAS-2 tool was used for quality assessment. Our results demonstrated that WDT presented a pooled accuracy of 0.88 (95% CI: 0.86-0.90), with a sensitivity of 0.79 (95% CI: 0.70-0.87), specificity of 0.80 (95% CI: 0.60-1.00), PLR of 5.87 (95% CI: 2.49-13.88), NLR of 0.25 (95% CI: 0.13-0.51), DOR of 23.39 (95% CI: 3.45-158.71), and SROC of 0.75. Notably, WDT provided best detection for 3 days surrounding ovulation. Ring-type device, the use of multi-physiological parameters and the random forest algorithm method improved efficiency for WDT in the detection fertility window. Overall, WDT holds promise for fertility window tracking and could offer tentative support for optimizing pregnancy planning and monitoring women’s reproductive health.

## Introduction

Pregnancy management remains a global health priority, reforming the trajectory of individuals and families, as well as a human population issue. A well-planned pregnancy with appropriate preconception preparation^1^ (e.g., folic acid supplementation^2^, preconception counseling^3^) and evidence-based pregnancy care^4^ significantly reduces the risk of gestational complications. However, the annual number of unintended pregnancies increased from 80 million in 1994 to 121 million in 2019. Among them, over 61% of unintended pregnancies end in abortion^5,6^, which is associated with long-term infertility risks in females^7^. In addition, unplanned pregnancies are significantly linked with adverse maternal and neonatal outcomes^8^, including low birth weight^9^, prematurity^9^, and developmental disadvantages^10,11^. It also imposes a costly burden on the health care system^12,13^. In the United States, direct medical costs due to unplanned pregnancies are projected to be 5 to 12.6 billion^12,14^, whereas the prevention of unintended pregnancies will save over 5.6 billion annually^14^. Therefore, it is critical to develop effective strategies to promote the planned conception and pregnancy.

The fertility window spans from 5 days before ovulation to 24 h after, based on the sperm and ovum viability^15–17^. Clinical studies showed that the probability of conception increases progressively during the fertile window and peaks on the day preceding ovulation^18,19^, and then rapidly declines thereafter^18,19^. A prospective longitudinal study suggested that avoiding unprotected intercourse during the fertile period only leads to 0.4 unintended pregnancies occurring per 100 women^20^. Therefore, accurately monitoring the timing of ovulation is essential for either successful fertilization or contraception. However, the fertile window is highly dynamic and personalized. Although ovulation typically occurs between days 10 and 17 of the menstrual cycle, only 30% of women ovulate entirely within this period^15^, underscoring the need for precision strategies to personalize ovulation detection.

Interestingly, the fertility window is intricately fine-tuned by cyclic alternations of estradiol, progesterone, luteinizing hormone (LH), and follicle-stimulating hormone (FSH)^21^. These hormonal dynamics contribute to biphasic rhythms and physiological shifts in females. For instance, basal body temperature (BBT) elevates by 0.3 °C to 0.7 °C in the post-ovulatory luteal phase owing to the progesterone-derived thermoregulatory effects^22^. Hormonal rhythms also increase resting heart rate (HR) by approximately 2.5% and decrease approximately 2.5% vagally mediated heart rate variability (HRV) in the luteal phase^23,24^. Meanwhile, lifestyle factors, like night shift^25^, psychological pressure^26^, and intensive exercise^27^, may disrupt endogenous hormone homeostasis and thereby affect the timing and regularity of the fertility window. Those nuanced but substantial fluctuations during the menstrual cycle raise the possibility of real-time tracking and analysis of physiological trajectories for fertility window detection^28,29^.

Wearable digital technology (WDT) integrates wearable hardware devices with digital components, including data processing algorithms and mobile apps for health assessment, alarming, and clinical care^30^. They typically incorporate one or multiple biological sensors to monitor real-time physiological signals, such as HR, body temperature (BT), blood pressure (BP), oxygen saturation (SpO_2_), and track physical activities and sleep patterns^31^. With its advancement for continuous non-invasive monitoring of physiological parameters, WDT has been widely adopted in medical practice, including for detecting epilepsy during seizures^32^, asymptomatic cardiovascular diseases^33^, and rapid identification of SARS-CoV-2 infection^34^. However, the current application of WDT in female health is still in the early stages, especially for the menstrual cycle and fertility window.

In this systematic review and network meta-analysis (NMA), we comprehensively synthesize current studies applying WDT to monitor the fertility window and menstrual cycles. Our primary outcome is to evaluate their accuracy, sensitivity, and specificity. We further employed Bayes network analysis to compare the performance of various device configurations, physiological parameters, algorithm methods, and detection intervals. Our study highlights the emerging clinical value of WDT in women’s reproductive health, providing a non-invasive and data-driven approach to personalized pregnancy plans and birth control.

## Results

### Characteristics of the studies

**Fig. 1:**
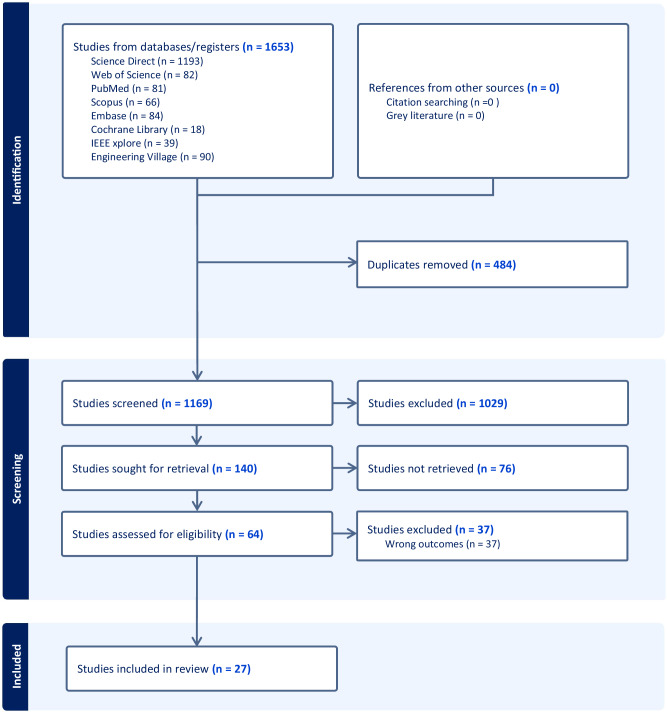
PRISMA flowchart of study search and selection strategy. We retrieved 1653 records from 8 databases, and 140 potential full texts were retrieved and assessed for their eligibility. Among them, 76 studies are conference abstracts or patent abstracts, and the full text or crucial demographic information and data for synthesis were unavailable. After the full-text screening, 27 studies were included for our Bayesian network meta-analysis. The figure was created by Microsoft Office PowerPoint 2021.

We retrieved 1653 records from 8 databases, and 140 potential full texts were retrieved and assessed for their eligibility. Among them, 76 studies were conference abstracts or patent abstracts, and the full text or crucial demographic information and data for synthesis were unavailable. After the full-text screening, 27 studies were included, involving 6244 participants and 14288 menstrual cycles in this systematic review and NMA (Fig. 1). The characteristics of the included studies using the WDT and conventional methods were stated in Table 1 and Table 2, respectively.

**Table 1:** Characteristics of the included studies using wearable digital technology for detecting the menstrual cycle and fertility window

Author	Region	Study design	Participant/Cycle	Age	BMI	Health status	Menstruation	Standard	Time	Analytical parameter	Algorithm	Outcome
Niggli A.,2023	Switzerland	Cross-sectional	61/205	26.5 ± 4.2	22.1 ± 2.9	healthy	regular	LH urine test	night	WST, HR, HRV, RR, SP	random foreset model	①②③④⑤⑥⑦
			3268/6081	32.5 ± 4.3	25.5 ± 6.27	healthy	regular			WST, HR, HRV, RR, SP		
Yu J.L.,2022	China	Cross-sectional	89/305	32.00 (27.00, 35.00)	20.82 (19.33, 22.59)	healthy	regular	ultrasound, serum PdG levels	night	WST, (WST + HR)	linear mixed model	①③④⑤⑥⑦
			25/77	28.00 (25.00, 31.00)	20.70 (19.29, 23.51)	healthy	irregular			WST, HR		
Zhu T.Y.,2021	Switzerland	Cross-sectional	57/193	26.7 ± 4.2	22.5 ± 3.6	healthy	regular+irregular	LH urine test	night	WST	linear mixed model	①②③④⑤⑥⑦
Goodale B.M.,2019	Switzerland	Cross-sectional	193/708	33.02 ± 3.68	22.70 ± 3.40	healthy	regular	LH urine test	night	WST, HR, RR	random forest model	①②③④⑤⑥⑦
B.S.H.,2022	US, UK	Cross-sectional	80/205	32	NA	healthy+PCOS+hypothyroid	regular+irregular	overnight vaginal temperature	night	WST	“spring- loaded beam” method	①②③④⑤⑥⑦
Shilaih M.,2018	Switzerland	Cross-sectional	194/793	33.66 ± 3.86	22.97 ± 3.68	healthy	regular	LH urine test	night	WST	linear mixed model	①③④⑤⑥⑦
Li Q.,2024	China	Cross-sectional	93/93	NA	NA	healthy	regular	LH urine test	night	FST	NA	①③④⑤⑥⑦
Gombert-Labedens M., 2024	US	Cross-sectional	111/111	18-52	NA	healthy	regular	LH urine test	night	FST	cosinor curve	①③④⑤⑥⑦
Luo L.,2020	US	Cross-sectional	22/39	NA	NA	NA	NA	LH urine test	night	BT	hidden Markov model	①③④⑦
Prasannan R.,2020	India	Cross-sectional	30/30	30-40	NA	NA	NA	NA	NA	BT	support vector machine	①③④⑦
Regidor P.A.,2018	Germany	Cross-sectional	158/470	18-45	18.2-36.9	healthy+PCOS	regular+irregular	LH, FSH, E2, PdG urine test	all day	BT	NA	①③④⑦
Weiss G.,2022	Austria	Cross-sectional	74/74	20-40	18.5-30	primary/secondary infertility+frozen embryo transfer+healthy	regular	ultrasound, serum progesterone levels, urine LH test	all day	BT	NA	①③④⑦
Sato D., 2024	Japan	Cross-sectional	26/74	NA	NA	healthy	NA	LH urine test	night	BT	linear mixed model	①②③④⑦

Abbreviations: *BMI* body mass index, *BT* body temperature, *E2* estradial, *FSH* follicle stimulating hormone, *HR* heart rate, *HRV* heart rate variability, *LH* luteinizing hormone, *NA* not appliable, *RR* respiratory rate, *SP* skin perfusion, *PdG* pregnanediol, *US* United States, *UK* United Kingdom, *WST* wrist skin temperature, *FST* finger skin temperature, *PCOS* polycystic ovary syndrome.

① primary outcome 1: the accuracy of WDT, self-reported BBT, electronic hormone testing system and calendar estimation in detecting fertility window; ② primary outcome 2: the specificity, sensitivity, positive likelihood ratio, negative likelihood ratio, diagnostic odds ratio and SROC of WDT and self-reported BBT; ③ secondary outcome 1: the accuracy of WDT based on distal BT and proximal BT in detecting fertility window; ④ secondary outcome 2: the accuracy of WDT with BT-based algorithm and multi-parameter-based algorithm in detecting fertility window; ⑤ secondary outcome 3: the accuracy of WDT with algorithm of random forest model, linear mixed model and other AI models in detecting fertility window; ⑥ secondary outcome 4: the accuracy of different modality of WDT (bands and rings) in detecting fertility window; ⑦ secondary outcome 5: the accuracy of different interval of WDT in detecting fertility window.

**Table 2:** Characteristics of the included studies using conventional methods for detecting the menstrual cycle and fertility window

Author	Region	Study design	Participant/Cycle	Age	BMI	Health status	Menstruation	Standard	Time	Analytical parameter	Outcome
Zhu T.Y.,2021	Switzerland	Cross-sectional	57/193	26.7 ± 4.2	22.5 ± 3.6	healthy	regular+ irregular	LH urine test	night	BBT	①②
Luo L.,2020	US	Cross-sectional	22/39	NA	NA	NA	NA	LH urine test	night	BBT	①
Martinez A.,1992	Netherlands	Cross-sectional	88/210	NA	NA	NA	regular	ultrasound	night	BBT	①
Tabbaa S., 2024	US, Canada	Cross-sectional	9/12	NA	NA	healthy	regular	ultrasound, serum LH levels	night	BBT	①
Sato D., 2024	Japan	Cross-sectional	26/74	NA	NA	healthy	NA	LH urine test	night	BBT	①②
Guermandi E.,2001	Italy	Cross-sectional	101/101	31.8 ± 3.4	NA	NA	NA	ultrasound	night	BBT	①
Behre H.M.,2000	Germany	Cross-sectional	53/150	26	NA	healthy	regular	ultrasound	morning	urine LH + E3G	①
Pattnaik S.,2023	US	Cross-sectional	52/52	27.6 ± 4.1	NA	healthy	regular	PdG urine test 7 days after LH peak	morning	urine LH + E3G	①
Mu, Q.,2023	US	Cross-sectional	15/43	33.07 ± 4.50	23.57 ± 1.00	healthy	regular	CBFM	morning	urine LH, urine LH + E3G	①
Bouchard T. P.,2019	US	Cross-sectional	13/34	33.6 ± 6.4	NA	healthy	regular	LH urine test	morning	urine PdG	①
Wegrzynowicz, A. K.,2022	US	Cross-sectional	40/40	35	NA	NA	NA	PdG urine test	morning	urine FSH, E1G, LH, PdG	①
MacGregor E. A.,2005	UK	Cross-sectional	27/1122	43	NA	menstrual related migraine	NA	PdG, LH, FSH, E1G urine levels	morning	urine LH + E3G	①
Thakur R., 2020	India	Cross-sectional	360/360	NA	NA	NA	NA	NA	morning	urine LH	①
Barron M. L.,2018	US	Cross-sectional	42/219	NA	NA	NA	NA	LH urine test	morning & evening	urine LH + E3G	①
Mouriki. E,2019	Switzerland	Cross-sectional	34/34	NA	NA	healthy	regular	LH urine test	morning	Standard Days method	①
Johnson S.,2018	UK	Cross-sectional	768/768	32	26.67	healthy+PCOS+endometriosis	regular+ irregular	LH urine test	morning	73 calendar apps- cycle length,cycle variability,last menstrual period	①
Suman S., 2023	India	Cross-sectional	161/1685	21-43	NA	NA	NA	NA	NA	artificial neural network and multiple linear regression based on age, luteal phase, previous cycle of ovulation, previous length of menses	①

Abbreviations: *BBT* basal body temperature, *BMI* body mass index, *CBFM* Clearblue Fertility Monitor, *E1G* oestrone-3-glucuronide, *E3G* *PdG* pregnanediol, *FSH* follicle stimulating hormone, *LH* luteinizing hormone, *PCOS* polycystic ovary syndrome, *NA* not appliable, *US* United States, *UK* United Kingdom.

① primary outcome 1: the accuracy of WDT, self-reported BBT, electronic hormone testing system and calendar estimation in detecting fertility window; ② primary outcome 2: the specificity, sensitivity, positive likelihood ratio, negative likelihood ratio, diagnostic odds ratio and SROC of WDT and self-reported BBT.

For the included studies, the diagnostic accuracy comparison included 13 studies on WDT^35–47^, 6 studies on self-reported BBT^37,44,46,48–50^, 8 studies on the electronic hormone testing system^51–58^, and 3 studies on the calendar method by self-calculation or calendar apps^59–61^. Among them, 5 WDT studies^35,36,38,44,46^ and 2 self-reported BBT studies^44,46^ reported true/ false positive/negative accounts. Ovulation was confirmed using various standards: 12 studies used the urine LH test^36–38,41,44–47,53,59,60,62^, 2 studies were based on the urine pregnanediol (PdG) test^56,58^, 3 studies employed the urine multi-hormone test^40,54,55^, 3 studies used the ultrasound method^48,49,52^, 3 studies applied ultrasound with serum hormone levels^42,43,50^, one study referred to overnight vaginal temperature^35^, and 2 studies did not specify the standard approach^57,61^. Among the 13 studies on WDT, in the aspect of hardware design, 6 studies used wrist-wear devices (band form)^35,36,38,41,43,44^ and 2 studies used finger-wear devices (ring form)^45,47^, where the rest studies applied in-ear devices^37^, lower abdomen band^39^, vaginal ring^40^, axillary sensor^42^, and chest sensor^46^, respectively. In the aspect of bio-signals, 10 cohorts collected distal or proximal body temperatures^35,37,39–47^, and 5 cohorts acquired multi-parameters, like BBT, HR, HRV, respiratory rate (RR), and skin perfusion (SP)^36,38,43,47^. In the aspect of algorithms for prediction, all WDTs were based on artificial intelligence (AI), where 2 studies applied random forest (RF) models^36,38^, 4 studies applied linear mixed models (LMM)^41,43,44,46^, and 7 studies applied other AI model methods^37,39,40,42,45,47,63^.

### Risk of bias assessment

In quality assessment, most studies included in our analysis presented a low risk of bias in patient selection and clinical measurements (Supplementary Fig. 1a, b), except for one study that lacked a clear definition of the study population and enrollment^61^. In the aspect of reference standard, most of the studies presented an unclear risk of bias since these studies referred to urine hormone tests, which may detect varied percentages of LH surges with urinary LH kits of different manufacturers^64^, and 3 studies did not report the reference standard^39,57,61^. 6 studies presented a low risk of bias for they applied transvaginal ultrasonography, or in combination with serum hormone levels as the gold standard to determine ovulation^42,43,48–50,52^. One study was ranked as high risk since it referred to overnight vaginal temperature, which may not be reliable as a BBT method^35^. In terms of flow and timing, all studies presented an unclear risk of bias since not all the participants or cycles were included in the analysis. Publication bias was evaluated using six studies reporting the diagnostic odds ratio (DOR) of WDT^36,38,44,46,63^. The funnel plot exhibited an asymmetric distribution, suggesting potential publication bias (Supplementary Fig. 1c). Moreover, the quality of the evidence on WDT in detecting ovulation was graded as ‘low’, which was further downgraded to ‘very low’ due to serious risk of bias, inconsistency, and imprecision. (Supplementary Table 1**)**.

### Sensitivity analysis

Sensitivity analysis showed that omitting Yu J.L.’s study (cohort 2)^43^ reduced the heterogeneity in a multi-parameter group (*I*^2^ = 0%) and increased the pooled accuracy to 0.91 (95% CI: 0.90–0.92) (Supplementary Fig. 1d), which may be due to this study enrolling participants with irregular cycles. For other outcomes, no study included or excluded impacted the overall result estimation; thus, we retained the results as they were.

### WDT outperforms conventional body temperature measurement and calendar methods in fertility window detection

Currently, there are several methods commonly used for detecting ovulation and menstrual cycles, including calendar estimation, self-measured BBT, and electronic hormone testing systems. Therefore, we first evaluated the performance of WDT in detecting female fertility windows, in comparison with the traditional methods (Supplementary Tables 2 and 3).

**Fig. 2:**
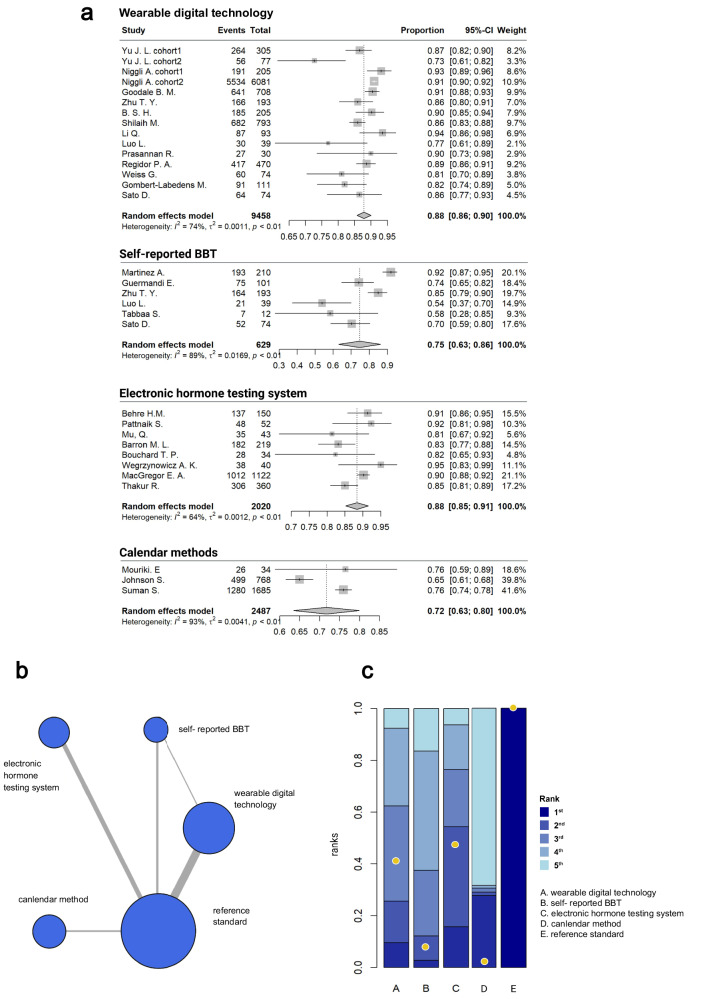
Comparison of wearable digital technology (WDT) and other methods in detecting the fertility window. **a** Forest plot and **b** Network plot of the pooled accuracy of WDT and other methods in detecting the fertility window. Edge thickness was proportional to the number of direct comparisons. Node sizes were proportional to the sample size. **c** Network meta-analysis ranking of the pooled accuracy of WDT and other methods for fertility window detection. Bars represented the ranking probability. Deeper blue represented a higher ranking. Among the bars in the same color, the length of the bar was proportional to its possibility in this ranking. Nodes represented the rank of surface under the cumulative ranking curve (SUCRA). A higher position of the node represented a higher ranking. Abbreviation: BBT basal body temperature. The figures were created by R software 4.1.0 (R Statistical Computing) using R package *metafor* and *gemtc*.

WDT demonstrated a pooled accuracy of 0.88 (95% CI: 0.86–0.90) for fertility window detection. Notably, it outperformed the self-reported BBT and calendar estimation, which showed a pooled accuracy of 0.75 (95% CI: 0.63–0.86) and 0.72 (95% CI: 0.63–0.80), respectively (Fig. 2a). Furthermore, in the comparison between WDT and self-reported BBT method, WDT yield pooled sensitivity and specificity of 0.79 (95% CI: 0.70–0.87) and 0.80 pooled (95% CI: 0.60–1.00), respectively, alongside a pooled positive likelihood ratio (PLR) of 5.87 (95% CI: 2.49–13.88), 0.25 negative likelihood ratio (NLR, 95% CI: 0.13-0.51), and 23.39 diagnostic odds ratio (DOR, 95% CI: 3.45-158.71), with the area under the summary receiver operating characteristic (SROC) curve of 0.752 (Supplementary Fig. 2a–c and Supplementary Fig. 3). By contrast, BBT-based studies exhibited a lower pooled sensitivity of 0.45 (95% Cl 0.01–0.90) and a pooled specificity of 0.73 (95% CI: 0.59–0.88), with PLR of 1.45 (95% CI: 0.35–5.93), NLR of 0.69 (95% CI: 0.25–1.86), DOR of 2.20 (95% CI: 0.20–24.29), achieving only a SROC of 0.368 (Supplementary Fig. 2a–c and Supplementary Fig. 3). Similarly, the network ranking and surface under the cumulative ranking curve (SUCRA) also indicated that WDT was superior to the BBT method (Supplementary Fig. 2d, e). Moreover, compared to the electronic hormone testing system, with a pooled accuracy of 0.88 (95% CI: 0.85–0.91), WDT presented a similarly pooled accuracy in detecting the fertility window. The data were further aligned with the network ranking and SUCRA analysis, where the electronic hormone testing displayed the highest detection performance, followed by WDT, self-reported BBT, and calendar methods (Fig. 2c).

Moreover, to identify potential heterogeneity of our results, we further performed subgroup analyses according to three categories, including 1) the menstrual regularity in participants (regular, irregular, mixed, or unspecified cycles), 2) health status of the participants (mixed populations including participants with comorbidities, or studies with unspecified health status), and 3) the ovulation detection method (ultrasound with serum hormone levels, serum LH alone, or overnight vaginal temperature). There was a significant difference in WDT diagnostic accuracy between menstrual regularity groups (Chi-square test, *p* = 0.02), indicating that irregular cycles may diminish the performance of WDT in ovulation detection (Supplementary Fig. 4a). In contrast, WDT exhibited stable and comparable performance across either different ovulation reference standards or participant health status (Chi-square test, *p* = 0.13 and 0.74, respectively, Supplementary Fig. 4b, c). Collectively, these findings suggest that although menstrual regularity remains a critical determinant of predictive reliability, WDT demonstrates consistent performance regardless of health background or ovulation validation method.

### WDT enables the detection of two-to three-day window surrounding ovulation

Instead of capturing the exact date of ovulation, WDT generally estimates a time interval surrounding the ovulation date, with a different duration depending on device design and computational method. Our analysis showed that the detection performance of WDT for the precise date of ovulation was relatively low, presenting a pooled accuracy of 0.56 (95% CI: 0.00–1.00) for the specific ovulation day, and 0.61 (95% CI: 0.53–0.70) for ±1 day surrounding ovulation. Moreover, WDT exhibited comparable performance for detecting ±2 days with accuracy of 0.90 (95% CI: 0.88–0.93) and ± 3 days with accuracy of 0.88 (95% CI: 0.84–0.92) surrounding ovulation, whereas a slightly lower pooled accuracy of 0.85 (95% CI: 0.80–0.91) was detected to detect ovulation 5 days in advance. Consistently, network ranking and SUCRA analysis revealed that the detection accuracy 2 to 3 days surrounding ovulation exceeded detecting ±1 day or the exact day of ovulation. Moreover, the interval from 5 days in advance to ovulation day was also suboptimal for WDT detection (Supplementary Fig. 5 and Supplementary Table 4).

### Body temperature is the core biometrics signal for the fertility window detection

Wearable devices are equipped with multiple biosensors to capture diverse physiological signals. We next examined the contribution of different physiological inputs in determining ovulation detection (Supplementary Table 5**)**.

**Fig. 3:**
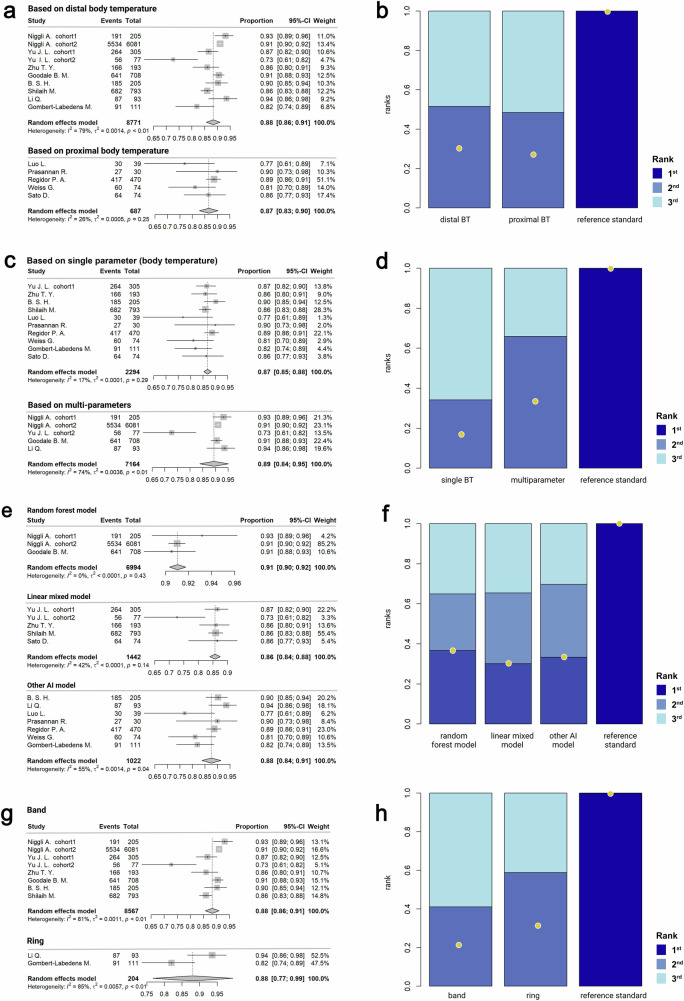
Comparison in the accuracy of the designs of wearable digital technology (WDT) in detecting the fertility window. **a**,**b** **a** Forest plot and **b** Network meta-analysis (NMA) ranking of the pooled diagnostic accuracy of WDT using different body temperature measurement in detecting the fertility window. **c**,**d** **c** Forest plot and **d** NMA ranking of the pooled diagnostic accuracy of WDT employing different parameters for fertility window detection. **e**,**f** **e** Forest plot and **f** NMA ranking of pooled accuracy of WDT based on different algorithms in detecting the fertility window. **g**,**h** **g** Forest plot and **h** NMA ranking of pooled accuracy of digital bands and rings in fertility window detection. In the NMA ranking plots, bars represented the ranking probability. Deeper blue represented a higher ranking. Among the bars in the same color, the length of the bar was proportional to its possibility in this ranking. Nodes represented the rank of surface under the cumulative ranking curve (SUCRA). A higher position of the node represented a higher ranking. Abbreviation: artificial intelligence (AI), body temperature (BT). The figures were created by R software 4.1.0 (R Statistical Computing) using R package *metafor* and *gemtc*.

Body temperature rhythmically fluctuates due to the thermoregulatory effects of progesterone throughout the menstrual cycle^22^. We found that WDT used distal BBT as a major index for detection, including wrist skin temperature (WST) and finger skin temperature (FST), showed a pooled accuracy of 0.88 (95% CI: 0.86–0.91). Similarly, studies applied proximal BBT for detection, including BBT on sites of acoustic meatus, low abdomen, vagina, axillar, and chest, achieved a pooled accuracy of 0.87 (95% CI: 0.83–0.90) (Fig. 3a). Consistently, network ranking and SUCRA analysis suggested both distal BBT and proximal BBT exhibited a comparable effect in fertility window detection (Fig. 3b).

### Multi-parameter and advanced algorithms improve the accuracy of ovulation detection

Apart from BT, WDTs enable simultaneous measurement of multiple physical signals, including WST, HR, HRV, RR, and SP. Compared with the single-BBT method with the pooled accuracy of 0.87 (95% CI: 0.85-0.88), multi-parameter-based detection yielded an improved pooled accuracy of 0.89 (95% CI: 0.84–0.95) (Fig. 3c). Consistently, network ranking and SUCRA ranked the multi-parameter design outperforming the single BBT design in the detection accuracy (Fig. 3d), indicating the clinical value of multidimensional bio-signal tracking for monitoring the fertility window.

To process multidimensional datasets, WDT incorporates sophisticated digital platforms with advanced algorithms, including conventional approaches, such as linear mixed models (LMM), to cutting-edge AI techniques like random forests (RF). Therefore, we subsequently interrogate the optimal computational method (Supplementary Table 5**)**. Our analysis suggested WDT based on LMM achieved a pooled accuracy of 0.86 (95% CI: 0.84-0.88). Furthermore, AI-based models, such as support vector machine^36,39,43,45^ and hidden Markov model^37^, exhibited positive effects to improve the pooled accuracy (0.88, 95% CI: 0.84–0.91). Of note, WDT with RF model achieved the highest accuracy of 0.91 (95% CI: 0.90–0.92) (Fig. 3e). Consistently, our network ranking and SUCRA analysis showed the order of probability was RF model > other AI model > LMM (Fig. 3f). Taken together, our data suggested that applying WDTs integrating with advanced AI algorithms may enhance the precision and robustness of fertility window detection.

### The design modality of WDT affects the accuracy in ovulation detection

WDTs are engineered in diverse forms, including wrist-worn bands, finger rings, and skin-adhered patches. Therefore, we subsequently evaluated how hardware design influences the ability of WDTs in fertility window monitoring. Among existing studies, only band and ring-type devices were utilized, with no alternative WDT forms reported. Our comparative analysis revealed band- and ring-type devices showed equivalent detecting performance, presenting pooled accuracy of 0.88 (95% CI: 0.86–0.91) and 0.88 (95% CI: 0.77–0.99), respectively (Fig. 3g). Interestingly, network ranking and SUCRA analysis positioned the ring-type devices as slightly superior to the band in detective performance (Fig. 3h), which is possibly due to their stable positioning and consistent skin contact.

## Discussion

Our study presents the first systematic review and Bayesian network meta-analysis to evaluate the effects of WDT on the detection of the menstrual cycle and fertility window in women. Despite current studies exhibiting considerable heterogeneity in physiological parameters acquisition, algorithm method, and device form, WDTs demonstrated reliable accuracy to monitor the fertility window compared to self-reported BBT and calendar-calculation methods. Moreover, our findings suggest that compact wearable formats, integrated with multiparameter monitoring and advanced AI-based algorithms, may further enhance predictive accuracy.

The menstrual cycle and fertility window constitute a core physiological axis shaping women’s lifelong health. Real-time and personalized fertility window tracking may not only support conception and contraception but also serve as a potential early signal for detecting reproductive disorders. However, traditional methods for ovulation prediction and menstrual tracking are limited by their accuracy, accessibility, and usability. For instance, though transvaginal ultrasound is the gold standard for ovulation tracking, it requires clinical visit^65^. The calendar estimation method relies on subjective self-reported cycle history and thus is vulnerable to recall bias^66,67^. The urinary hormone test typically needs repetitive testing, limiting its cost-effectiveness and user adherence. Our results highlighted that WDTs emerge as promising tools for detecting fertility windows in a personalized and real-time manner. WDTs outperformances over ordinary calendar methods with noticeably higher sensitivity (0.79 vs. 0.45) and SROC (0.75 vs. 0.37) and present comparable accuracy with hormone-based methods. In terms of detection intervals, WDT generally presented a high predictive value when identifying a fertility window within ± 3 days surrounding ovulation. However, given the viability of sperm (up to 5 days) and oocytes (about 24 h after ovulation)^40,68^, the current predictive interval of WDT cannot perfectly align with the biological fertility window. Further WDT development should aim to optimize the prediction time window from 5 days before to 24 h post-ovulation, which is essential to minimize the unintended conception and enhance pregnancy planning in clinical practice.

Alternations in BBT during the menstrual cycle are a well-established physiological phenomenon driven by the thermogenic effects of progesterone during the luteal phase. In our study, BBT still serves as the core parameter detected by WDT to provide the most reliable signal for improving predictive accuracy than the self-reported BBT method (0.87 (95% CI: 0.85–0.88) vs. 0.75 (95% CI: 0.63–0.86)). Although WDTs typically measure skin temperature, which may be affected by environmental factors or physical activity^69^, several studies have shown that continuously monitored skin temperature can linearly reflect core body temperature^70^. Of note, all the current WDTs employed the nocturnal BBT for the prediction, which can largely minimize environmental disturbance. Interestingly, we found FST and WST presented similarly pooled detection accuracy (0.88 (95% CI: 0.77–0.99) vs. 0.88 (95% CI: 0.86–0.91)), whereas FST presented higher network and SUCRA ranking, which may be attributed to the improved comfort and stability of ring-type devices for continuous BBT measurement.

Except for BBT, growing evidence suggests the alternations RR and HR, and decreases in HRV after ovulation^71,72^. These physiological shifts are mainly driven by either vague nerve-mediated or hormone-induced sympathetic changes and increased metabolic rate. Our data found inclusion of those parameters into algorithms could slightly improve the accuracy of WDT in detecting fertility windows. As shown, WDT integrating BBT and other physical parameters (solo or in combination of HR, HRV, RR, SP) outperformed those only based on BBT in detecting accuracy (0.89 (95% CI: 0.84–0.95) vs. 0.87 (95% CI: 0.85–0.89), which was confirmed by higher ranking of WDT with multiple parameters in network and SUCRA ranking. Taken together, although these variations are subtler than BBT changes, they may still enhance fertility prediction accuracy when integrated into multi-parameter algorithms. It further underscores the advantages of WDT in fertility window management, as they can trace a spectrum of physiological signals. Interestingly, it has been reported that a WDT-equipped chemical nano-biosensor is available for testing estradiol levels in body fluid^73^. This advancement suggests a promising future in which WDT may integrate microfluidics technology for detecting trace-level hormones, thereby offering more precise fertility tracking and personalized care.

Our study has several limitations. First, heterogeneity in the definition of ovulation varied across enrolled studies. Although the subgroup analysis on the accuracy of WDT with different reference standards showed comparable results, most studies estimated ovulation using serum LH levels and lacked validations of the gold standard method of ultrasound, which may impair the reliability and comparability. Second, although no significant effects were observed for ovulation detection standards, methodological inconsistencies and small sample sizes across studies may limit the generalizability of our findings. In our results, menstrual regularity was a key determinant of heterogeneity, and WDT was reported to exhibit lower detection accuracy in the irregular menstruation populations, implying that irregular cycles remain a challenge for WDT-based ovulation prediction. Further studies with rigorous design are needed to validate the capability of WDT in precisely predicting ovulation, especially in those with irregular cycles. Moreover, some cohorts we included in this analysis containing both healthy women and patients with reproductive disorders, such as Polycystic ovary syndrome (PCOS)^35,40^, hypothyroidism^35^, and infertility^42^, which may lead to a confounding factor. Further clinical study is urgent to assess the performance of WDT in women with irregular menstrual cycles. Nevertheless, there was comparable diagnostic accuracy of WDT between the two groups in our subgroup analysis, the impact of comorbidities on the diagnostic performance of WDT should be further investigated in future studies to provide personalized ovulation prediction. Furthermore, the absence of participant demographics in several studies limited our analysis to evaluate the impact of other potential confounders (e.g., age and body mass index (BMI)^35,37,39,40,42,45^. Third, although the probabilities and cumulative ranking suggested relatively optimal WDT designs, physiological parameters, and algorithmic approaches, these findings should be interpreted with caution. Most of pairwise comparisons revealed statistically significant differences only when compared to the reference standard, whereas direct comparisons between individual designs were largely non-significant. Therefore, the relative performance of different WDT configurations requires more rigorous head-to-head evaluation in future studies. Fourth, given the small number of included studies, the assumptions required for NMA may be unstable, leading to limited robustness of the findings. Moreover, publication bias and studies with a risk of bias may compromise confidence in the evidence, which may skew the overall distribution of observed values and overstate the actual diagnostic performance of WDT in the clinical practice. Together with the very low Grading of Recommendations, Assessment, Development and Evaluations (GRADE) rating of the certainty of the evidence, the accuracy of WDTs in menstrual tracking and fertility management should be interpreted with prudence. Importantly, large-scale cohorts with robust designs are warranted to rigorously validate the results. Importantly, the WDT detection interval with high accuracy does not optimally cover the biological fertility window. Future iterations should prioritize matching the prediction with the physiological fertility window, thereby improving clinical utility for both pregnancy planning and conception avoidance.

In summary, our systematic review and NMA demonstrate that WDTs may serve as promising tools for detecting ovulation within 3 days before and after ovulation. The integration of BBT with additional physiological signals into advanced AI-based, multi-parameter algorithms may further enhance detection accuracy. With the development of digital health and biosensing technologies, WDTs hold the potential as next-generation, non-invasive approaches for personalized care in women’s reproductive health.

## Methods

### Search strategy and selection criteria

Eight electronic databases (PubMed, Web of Science, Embase, CINAHL, Scopus, Science Direct, IEEE Xplore, EI village) were systematically searched for articles published until January 1, 2025. Details of the search strategies are available in the **Supplementary Note (Searching strategy)** for all the databases searched.

Studies included participants of women of reproductive age to track the fertility window by means of calendar estimation, self-measured BBT, WDT, or electronic hormone testing systems. The index involved the use of WDT and other ovulation-tracking methods, including ① calendar estimation: evaluating ovulation based on historical cycle length. The ovulation day should be the 14th day before the first day of the next menstrual bleeding. ② self-reported BBT: evaluating ovulation based on the progesterone-induced thermogenesis in the post-ovulatory phase. The participants take body temperature immediately upon waking in a ‌consistent daily measurement time, and record in a BBT chart. The ovulation day should be the day with a temperature shift. ③ electronic hormone testing system: evaluating ovulation based on the urinary luteinizing hormone levels, which are tested by a strip and then analyzed by a mobile-mounted, app-connected home-based device, reporting a binary digital result or quantified hormone levels. The targeted conditions were the performance metrics (accuracy, sensitivity, specificity, PLR, NLR, DOR, and SROC) of the methods aforementioned in detecting fertility window, compared with a reference standard of ovulation. The gold standard for ovulation documentation is direct ultrasound visualization of an egg extruded from the ovary. Indirect validation included urinary progesterone excretion (PdG)^74^, the midcycle LH peak^75^, and serum progesterone levels^76^. Eligible studies included cross-sectional, case-control or cohort studies that were published exclusively in the English language were considered for inclusion. We excluded the studies with participants undertaking oral contraceptive pills since the BBT and hormone testing methods for ovulation determination are based on natural hormone fluctuations. The studies without full text or crucial demographic information were also excluded if the authors could not be contacted for additional information. This study was registered at PROSPERO (CRD42024601664) and subsequently amended to include a NMA to compare the diagnostic accuracy of different WDT designs, parameters, and algorithms, in addition to the original protocol. The results were reported following the PRISMA NMA guidelines^77^.

### Definition the detection interval

WDT-detected fertility windows varied across different studies. There were 2 studies reported on the detection of exact ovulation day^41,47^, 2 studies reported one day before to after ovulation (±1 day)^47,63^, 4 studies reported 2 days before to after ovulation (± 2 days)^38,44,47^, 5 studies reported 3 days before to after ovulation (± 3 days)^37,40,42,47,63^, and 5 studies reports 5 days in advance to ovulation until the ovulation day (-5 days to ovulation)^36,39,43,45^ (Supplementary Table 6). For each outcome, we only synthesized the highest accuracy to avoid dependency in effect sizes if the study reported multiple detection intervals.

### Outcomes

The primary outcomes were ① the accuracy of WDT, self-reported BBT, electronic hormone testing system and calendar estimation in detecting fertility window; ② the specificity, sensitivity, PLR, NLR, DOR, and SROC of WDT and self-reported BBT; Secondary outcomes were ① the accuracy of WDT based on distal BBT and proximal BBT in detecting fertility window; ② the accuracy of WDT with BT-based algorithm and multi-parameter-based algorithm in detecting fertility window; ③ the accuracy of WDT with algorithm of random forest model, linear mixed model and other AI models in detecting fertility window; ④ the accuracy of different modality of WDT (bands and rings) in detecting fertility window; ⑤ the accuracy of different interval of WDT in detecting fertility window.

Accuracy was defined as the percent of cycles correctly classified by a method (the number of cycles correctly classified/total number of cycles). Sensitivity was defined as the ability to detect a true ovulation (the number of cycles correctly classified as ovulatory/total true number of ovulatory cycles. Specificity was defined as the ability to detect a true anovulation (the number of cycles correctly classified as anovulatory/total true number of anovulatory cycles). PLR was defined as the ratio of the probability of true ovulation to the probability of a false ovulation (the number of cycles correctly classified as ovulatory/total true number of ovulatory cycles divided by the number of cycles incorrectly classified as ovulatory/total true number of anovulatory cycles). NLR was defined as the ratio of the probability of a false anovulatory to the probability of a true anovulatory (the number of cycles incorrectly classified as anovulatory/total true number of ovulatory cycles divided by the number of cycles correctly classified as anovulatory/total true number of anovulatory cycles). DOR was defined as the ratio of the PLR to the NLR, representing the overall discriminative power of the diagnostic method.

### Data analysis

Two independent authors (Y.S. and C.C.W.) screened each article (title, abstract, and full text) eligible for the review and extracted data comprising the following components: characteristics of the studies (first author, year, region), characteristics of the subjects (age, BMI, health status, menstruation regularity), characteristics of the index test (parameter, algorithm, window range, measured time), characteristics of the standard (method for ovulation determination), number of participants analyzed, number of menstrual cycles analyzed, account of each outcome, and mean and/or standard deviation (SD), or the range of the continuous data. A third reviewer (Y.W.) resolved discrepancies between the 2 reviewers. We contacted the authors of the studies to request clarification for incomplete data.

Risk of bias was assessed using the Quality Assessment of Diagnostic Accuracy Studies–Revised (QUADAS-2)^78^ in Review Manager 5.4. Two review authors (Y.S. and C.C.W.) assessed the risk of bias for each trial via the risk of bias tool from 4 aspects: patient selection, index test, reference standard, and flow and timing. We ranked each domain as either a ‘low’, ‘unclear’ or ‘high’ risk of bias, and the first three in terms of concerns regarding applicability. We discussed with a third review author (Y.W.) to resolve discrepancies. The certainty of the evidence was assessed using the GRADE approach for the outcomes^79^.

Data from independent cohorts within a study were synthesized into an outcome. Given the likelihood of increased inter-observation variance, a random-effects model was used to assess the pooled accuracy, sensitivity, specificity, PLR, NLR, and DOR. All statistical analyses were performed by using R version 4.1.0 (R Statistical Computing). Forest plots were created by using R package *metafor* and *meta*. Between-study heterogeneity was assessed using the Cochran *Q* statistic (*p* < 0.05 indicated heterogeneity), the between-study variance was assessed using *I*^2^, and the magnitude of between-study variation due to true differences in effect sizes rather than chance will be assessed using *I*^2^. *I*^2^ ≥ 50% was considered substantial heterogeneity^80^. For any observed substantial heterogeneity, the possible reasons for this were examined by subgroup analyses to explore whether the diagnosis performance was moderated by the reference standards, health status, and menstrual cycle regularity. Chi-square test was applied to compare the detection accuracy among subgroups, and *p* < 0.05 indicated a significant difference.

The NMA was performed by using R package *gemtc* for the accuracy, sensitivity and specificity of the different methods, and different designs and intervals of WDT for fertility window detection, comparing with reference standard. We first checked the underlying assumption of NMA. In principle, all participants were able to observe fertility windows and applied similar methods or designs to monitor the fertility window across the studies within each group. We therefore determined that the assumption of transitivity and homogeneity held^81^. For the consistency test, we performed node-splitting assessments to determine the association between the direct and indirect evidence. All *I*^2^ values = 0%, indicating consistency between the direct comparison, indirect comparison, and network pooled results. For outcomes without closed loops in the network, heterogeneity was assessed using *I*^2^, and all *I*^2^ = 0% indicated low heterogeneity. Therefore, the consistency model was applied to the studies. Based on the held assumptions, we summarized the geometry of each evidence network using network plots for each outcome. Markov chain Monte Carlo (MCMC) simulation and potential scale reduction factor (PSRF) were used to evaluate the convergence of iteration. The rare variations and stability of various plots, and the PSRF value of all estimated outcomes of approximately 1.00 indicated complete convergence, good iterative effect, and stable results of the model. The ranking probabilities of each detective method were generated according to the Bayesian approach. The cumulative ranking was evaluated by SUCRA using R package *meta4diag* and *INLA*. The larger the SUCRA the higher its rank among all available methods^82^.

Publication bias was assessed with the symmetry of Deeks’ funnel plot using R package *meta* and *ggplot2*^83^, and an asymmetric funnel plot indicated the presence of publication bias. Sensitivity analyses were conducted to assess differences in the pooled effect estimates using a random-effect model, after removing each study^84^.

## Supplementary information

Supplementary Information

## Data Availability

All data generated or analyzed during this study are included in this published article and its supplementary information files.
