# Supplementary material for: The diagnostic accuracy of wearable digital technology in detecting fertility window and menstrual cycles: a systematic review and Bayesian network meta-analysis
Source: NPJ Digit Med. 2026 Jan 24;9:139. doi: 10.1038/s41746-025-02320-8 (PMC12886881; doi:10.1038/s41746-025-02320-8)
Supplement: Supplementary file 1 — Supplementary Information [file 41746_2025_2320_MOESM1_ESM.pdf]

Supplementary Fig. 1

a

b

c

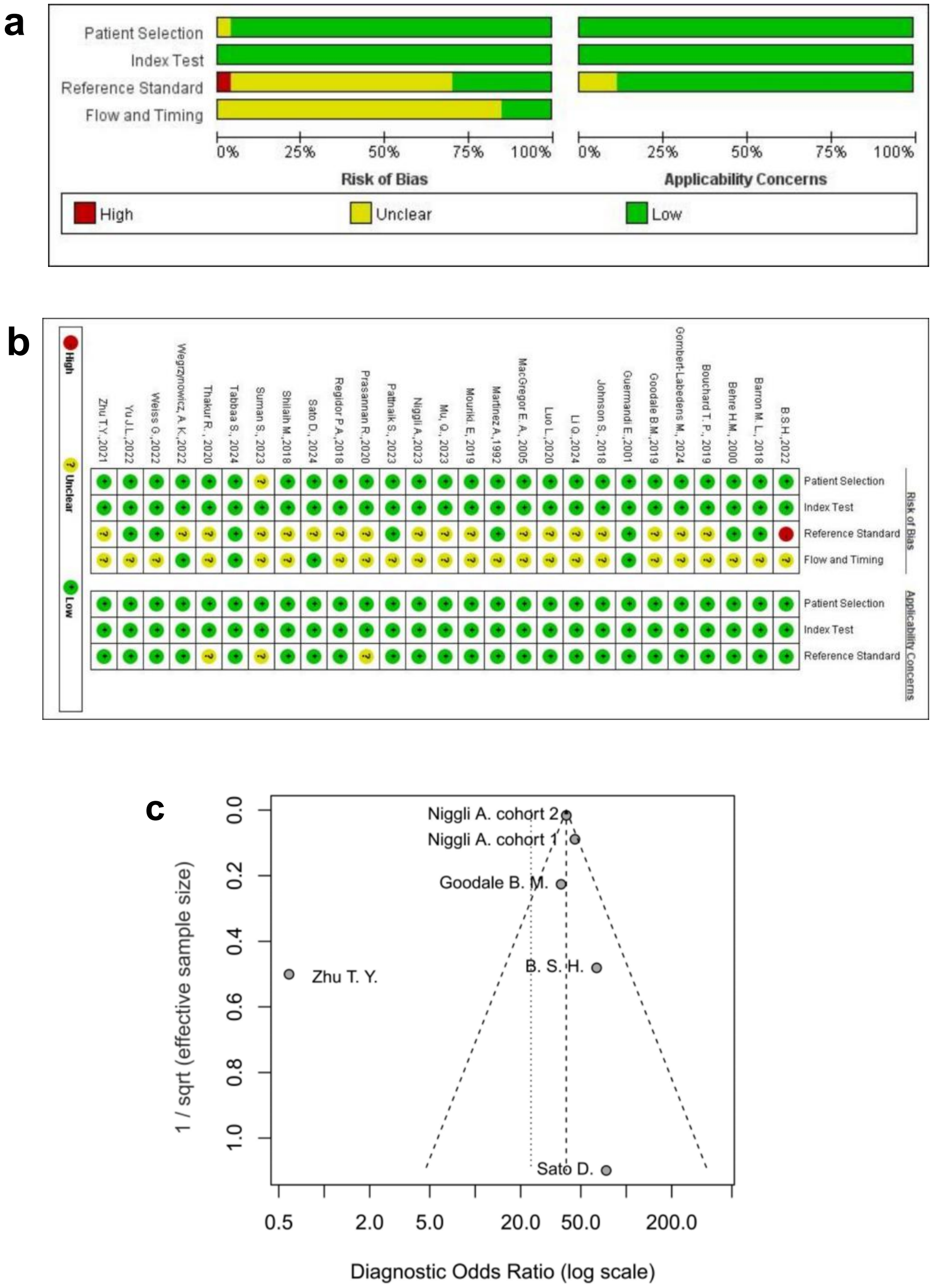

d

Study	Proportion	95%-CI	P-value	Tau2	Tau	I2
Omitting Niggli A. cohort1	0.88	[0.80; 0.96]	.	0.0057	0.0752	79%
Omitting Niggli A. cohort2	0.88	[0.80; 0.97]	.	0.0065	0.0807	81%
Omitting Yu J. L. cohort2	0.91	[0.90; 0.92]	.	0	0	0%
Omitting Goodale B. M.	0.89	[0.80; 0.97]	.	0.0066	0.0809	81%
Omitting Li Q.	0.88	[0.80; 0.96]	.	0.0055	0.0741	80%
**Random effects model**	**0.89**	**[0.84; 0.95]**	**.**	**0.0036**	**0.0597**	**74%**

0.6 0.8 1

Supplementary Fig. 1. Quality assessment and sensitivity analysis. (**a**). Risk of bias graph of the included studies. (**b**). Risk of bias summary of the included studies. (**c**). Deek's funnel plot of publication bias assessment of the pooled diagnostic odds ratio (log transformed) of WDT. (**d**). Forest plot of the sensitivity analysis of WDT based on multiple parameters in detecting fertility windows. The figures were created by Review Manager 5.4 and R software 4.1.0 (R Statistical Computing) using the R package *meta* and *ggplot2.*

Supplementary Fig. 2

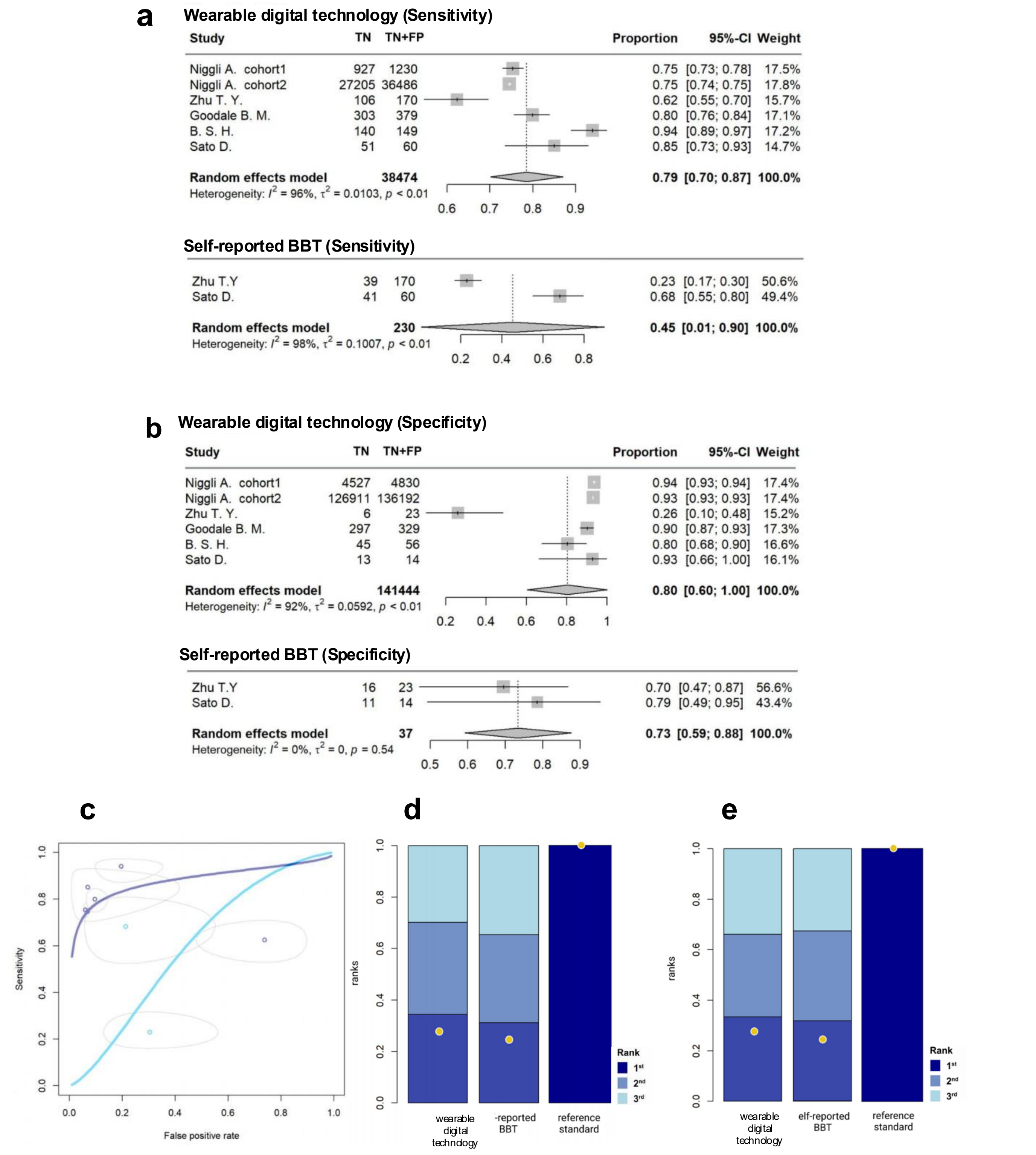

Supplementary Fig. 2. Comparison of the sensitivity and specificity of wearable digital technology (WDT) and self-reported basal body temperature (BBT). (**a**, **b**) Forest plot of (**a**) pooled sensitivity and (**b**) pooled specificity of WDT and self-reported BBT in fertility window detection. (**c**) Summary receiver operating characteristic (SROC) of WDT and self-reported BBT for fertility window detection. The dark blue and light blue curves represent the SROC of WDT and self-reported basal body temperature, respectively. (**d**, **e**) Network meta-analysis (NMA) ranking of (**d**) pooled sensitivity and (**e**) pooled specificity of WDT and BBT for fertility window detection. In the NMA ranking plots, bars represented the ranking probability. Deeper blue represented a higher ranking. Among the bars in the same color, the length of the bar was proportional to its possibility in this ranking. Nodes represented the rank of surface under the cumulative ranking curve (SUCRA). A higher position of the node represented a higher ranking. Abbreviation: wearable digital technology (WDT), basal body temperature (BBT). The figures were created by R software 4.1.0 (R Statistical Computing) using R packages *metafor*, *gemtc, meta4diag,* and *INLA*.

Supplementary Fig. 3

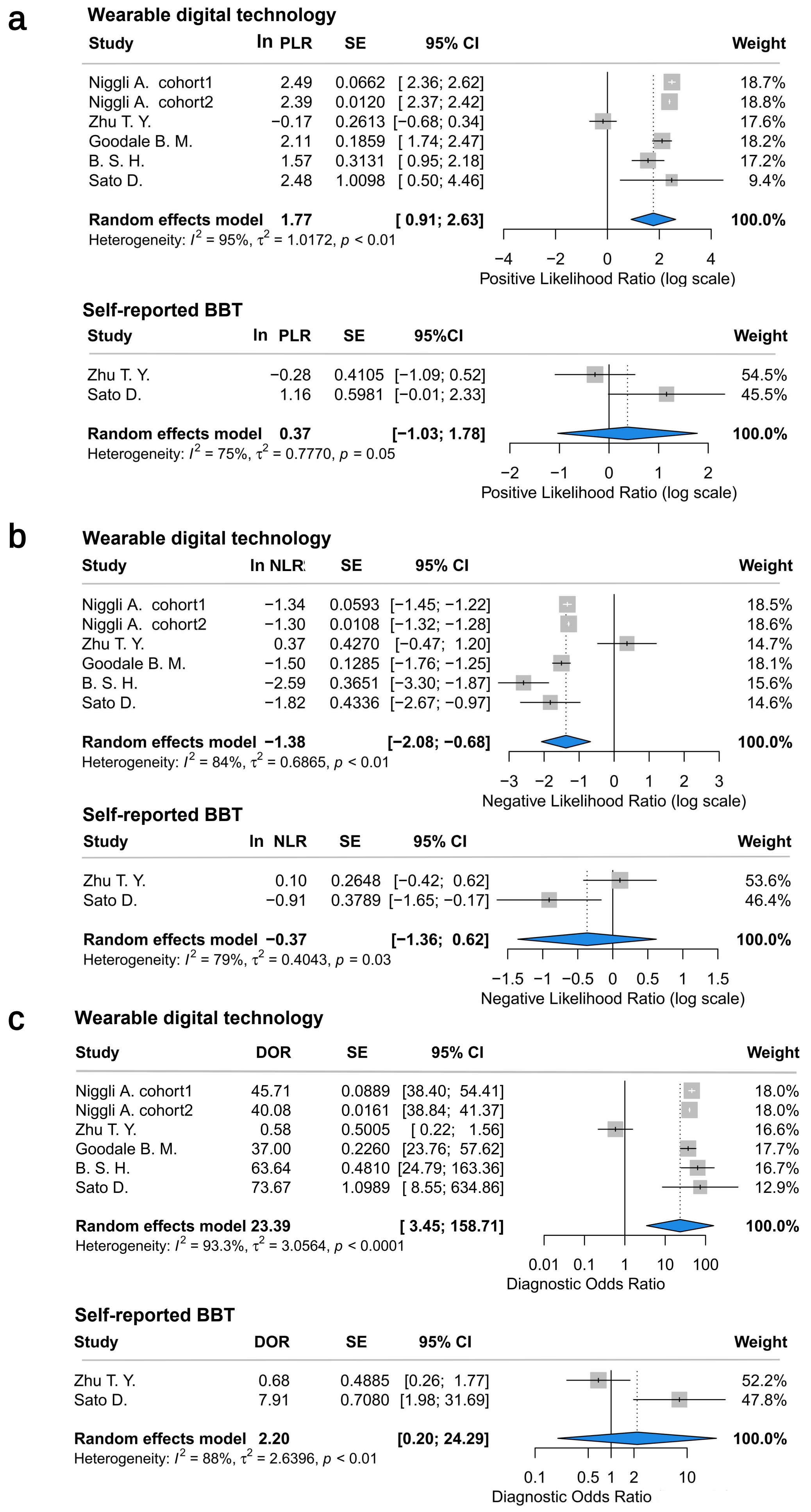

Supplementary Fig. 3 Comparison of the positive likelihood ratio (PLR), negative likelihood ratio (NLR), and diagnostic odds ratio (DOR) of WDT and self-reported BBT. (**a, b, c**) Forest plot of (**a**) pooled PLR (log transformed), (**b**) pooled NLR (log transformed), and (**c**) pooled DOR of WDT and self-reported BBT for fertility window detection. Abbreviation: wearable digital technology (WDT), basal body temperature (BBT). The figures were created by R software 4.1.0 (R Statistical Computing) using R packages *metafor* and *meta*.

Supplementary Fig. 4

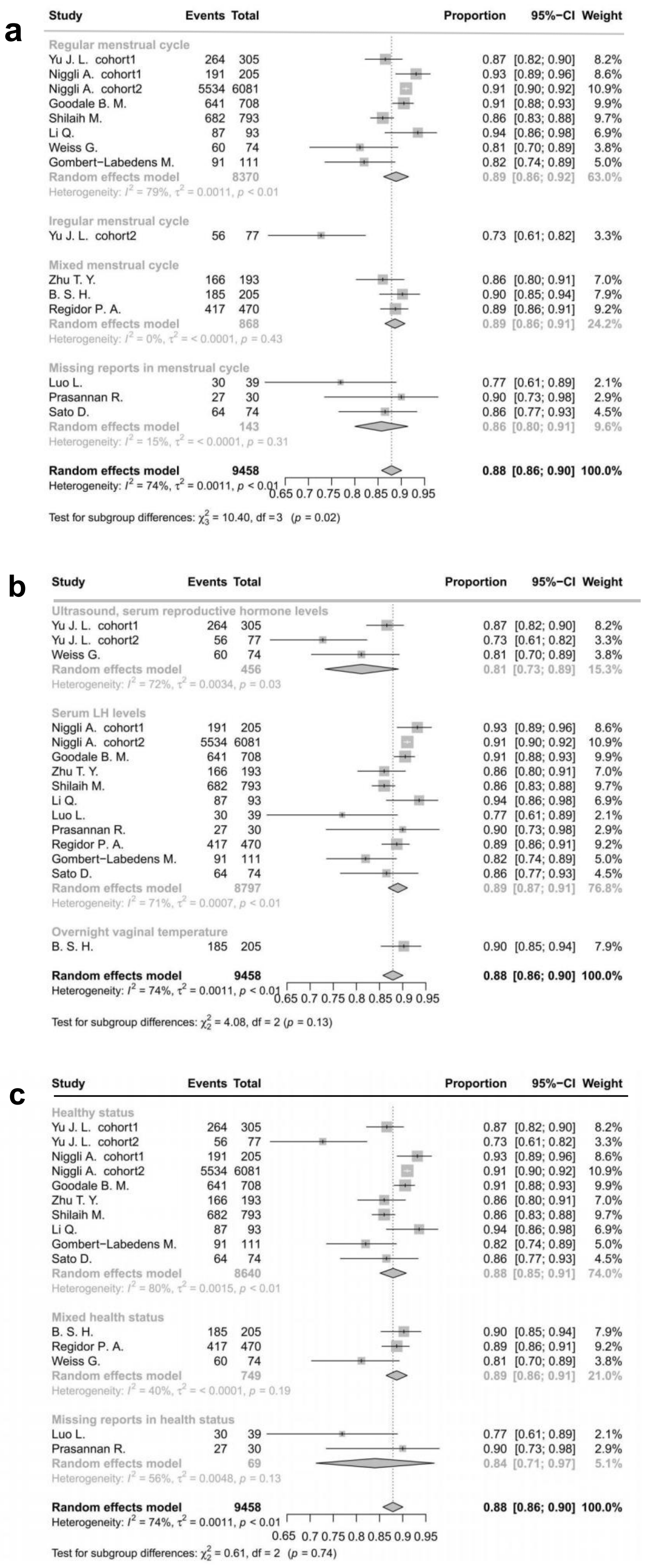

Supplementary Fig. 4. Accuracy of wearable digital technology (WDT) in detecting ovulation in subgroups with varied reference standards, health status, and menstrual cycle regularity. (**a, b, c**) Forest plot of the pooled accuracy of WDT for fertility window detection in subgroups with (**a**) varied cycle irregularities (the mixed status group contained participants with regular and irregular menstruation), (**b**) different healthy statuses (the mixed group contained healthy participants and patients with comorbidities, including polycystic ovary syndrome, hypothyroidism, infertility, and those who underwent frozen embryo transfer), and (**c**) varied reference standards. The figures were created using R software 4.4.1. The figures were created by R software 4.1.0 (R Statistical Computing) using R packages *metafor* and *meta*.

Supplementary Fig. 5

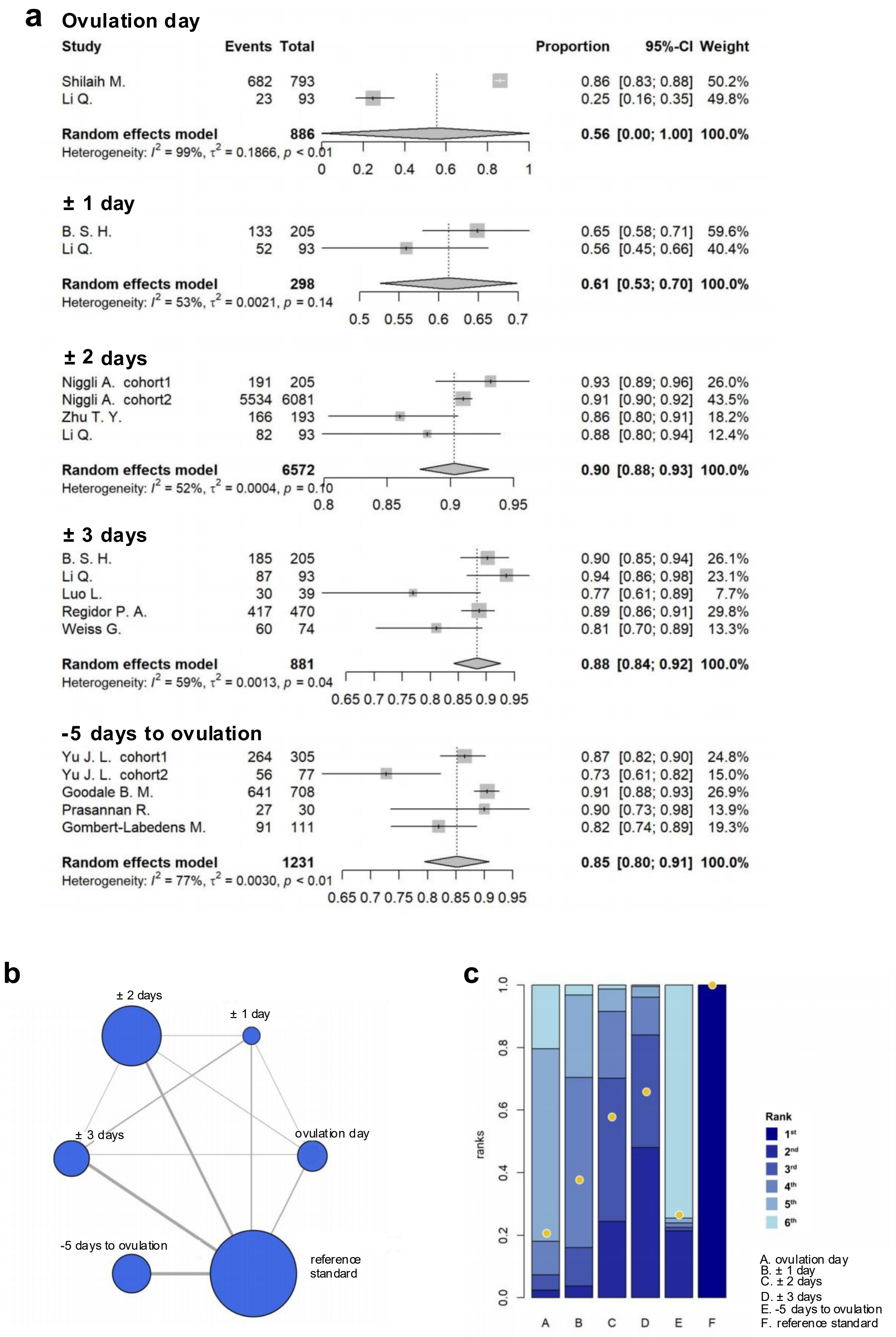

Supplementary Fig. 5 Accuracy of wearable digital technology (WDT) in detecting different fertility window intervals. (**a**) Forest plot and (**b**) Network plot, and (**c**) Network meta-analysis (NMA) ranking of the pooled accuracy of different intervals of WDT for fertility window detection. In the network, edge thickness was proportional to the number of direct comparisons. Node sizes were proportional to the sample size. In the ranking plot, bars represented the ranking probability. Deeper blue represented a higher ranking. Among the bars in the same color, the length of the bar was proportional to its possibility in this ranking. Nodes represented the rank of surface under the cumulative ranking curve (SUCRA). A higher position of the node represented a higher ranking. Abbreviation: Ovulation day: exact ovulation day, ±1 day: from one day before to after ovulation, ±2 days: from 2 days before to after ovulation, ±3 days: from 3 days before to after ovulation, -5 days to ovulation: 5 days in advance of the ovulation day. The figures were created using R software 4.4.1. The figures were created by R software 4.1.0 (R Statistical Computing) using R packages *metafor* and *meta*.

Supplementary Note

Searching strategy

Pubmed-31+50   WOS-37+45   Embase-53 + 31

((‘female’ OR ‘woman’) AND (‘device’ OR ‘sensor’ OR ‘sensing’ OR ‘biosensor’ OR ‘monitor’ OR ‘monitoring’ OR ‘smart’) AND (‘wearable’) AND (‘menstruation’ OR ‘menstrual cycle’ OR ‘menarche’ OR ‘menstrual period’ OR ‘mense’ OR ‘ovulation’ OR ‘ovulate’ OR ‘ovulatory’ OR ‘fertility’ OR ‘fertile’ OR ‘fertile window’ OR ‘LH’ OR ‘luteinizing hormone’ OR ‘luteal phase’)) NOT (‘review’ OR ‘meta-analysis’ OR ‘systematic review’)

((‘BBT’ OR ‘basal body temperature’ OR ‘calendar’ OR ‘urine test’ OR ‘mobile app’) AND (‘ovulation’ OR ‘ovulate’ OR ‘ovulatory’ OR ‘fertility’ OR ‘fertile’ OR ‘fertile window’) AND (‘detect’ or ‘predict’ or ‘monitor’) AND (‘accuracy’)) NOT (‘review’ OR ‘meta-analysis’ OR ‘systematic review’)

Science direct-588 + 605

(‘female’ OR ‘woman’) AND (‘device’ OR ‘sensor’) AND (‘wearable’) AND (‘menstrual cycle’) AND ‘research articles’ (article type)

(‘BBT’ OR ‘urine test strip’ OR ‘mobile app’) AND (‘ovulation’ OR ‘fertility’) AND (‘accuracy’) AND ‘research articles’ (article type)

Scopus-32 + 34

(‘female’ OR ‘woman’) AND (‘device’ OR ‘sensor’) AND (‘wearable’) AND (‘menstrual’ OR ‘‘ovulation’ OR ‘fertility’) AND (LIMIT-TO (DOCTYPE , "ar"))

(‘bbt’ OR ‘urine AND test AND strip’ OR ‘mobile AND app’) AND (‘ovulation’ OR ‘fertility’) AND (‘accuracy’) AND (LIMIT-TO (DOCTYPE , "ar"))

Cochrane Library-13 + 5

((‘female’ OR ‘woman’) AND (‘device’ OR ‘sensor’ OR ‘sensing’ OR ‘biosensor’ OR ‘monitor’ OR ‘monitoring’ OR ‘smart’) AND (‘wearable’) AND (‘menstruation’ OR ‘menstrual cycle’ OR ‘menarche’ OR ‘menstrual period’ OR ‘mense’ OR ‘ovulation’ OR ‘ovulate’ OR ‘ovulatory’ OR ‘fertility’ OR ‘fertile’ OR ‘fertile window’ OR ‘LH’ OR ‘luteinizing hormone’ OR ‘luteal phase’))

(‘BBT’ OR ‘urine test’ OR ‘mobile app’) AND (‘menstruation’ OR ‘menstrual cycle’ OR ‘menarche’ OR ‘menstrual period’ OR ‘mense’ OR ‘ovulation’ OR ‘ovulate’ OR ‘ovulatory’ OR ‘fertility’ OR ‘fertile’ OR ‘fertile window’) AND (‘accuracy’)

IEEE- 25+14, EI-73 + 17

(‘device’ OR ‘sensor’) AND (‘wearable’) AND (‘menstrual’ OR ‘‘ovulation’ OR ‘fertility’)

(‘mobile app’) AND (‘menstrual’ OR ‘‘ovulation’ OR ‘fertility’) AND (‘accuracy’)

Supplementary Table 1.
GRADE assessment for wearable digital technology in diagnosing ovulation

Outcome	No. of studies (No. of cycles)	Study design	Factors that may decrease certainty of evidence					Effect per 1,000 patients tested pre-test probability of 80%	Test accuracy CoE
			Risk of bias	Indirectness	Inconsistency	Imprecision	Publication bias		
**True positives** (cycles correctly classified as ovulatory)	6 studies 38474 cycles	cross-sectional (cohort type accuracy study)	serious	not serious	serious	serious	not serious	500 (443 to 551)	⊕◯◯◯
**False negatives** (cycles incorrectly classified as anovulatory)								133 (82 to 190)	Very low
**True negatives** (cycles correctly classified as anovulatory)	6 studies 141444 cycles	cross-sectional (cohort type accuracy study)	serious	not serious	serious	serious	not serious	294 (220 to 367)	⊕◯◯◯ Very low
**False positives** (cycles incorrectly classified as ovulatory)								73 (0 to 147)	

Sensitivity: 0.79 (95% CI: 0.70 to 0.87), Specificity: 0.80 (95% CI: 0.60 to 1.00), Prevalences: 63.3%

Supplementary Table 2.

League table of the comparison of wearable digital technology and other methods in the accuracy of detecting fertility window

**wearable digital technology**	-	-	-	-
0.493 (-4.152, 5.208)	**self-reported basal body temperature**	-	-	-
-1.118 (-11.560, 9.130)	-1.616 (-10.972, 7.511)	**electronic hormone testing system**	-	-
-2.070 (-10.308, 5.922)	-2.569 (-9.315, 3.964)	-0.958 (-7.523, 5.557)	**calendar methods**	-
-40.259 (-131.011, -11.168)	-40.838 (-131.674, -11.578)	-39.331 (-129.652, -9.875)	-73.417 (-244.430, -11.268)	**reference standard**

Data presented the log-transformed odds ratio (log OR) comparing the accuracy of detecting method in the column to that of detecting method in the row or reference standard. A positive value indicates the method in the column showed higher accuracy than the method in the row in detecting fertility window, whereas a negative value indicates the method in the column is less accurate than the method in the row.

Supplementary Table 3.

League table of the comparison of the sensitivity, specificity, positive likelihood ratio, negative likelihood ratio, and diagnostic odds ratio of wearable digital technology and self-reported basal body temperature

Sensitivity		
**wearable devices**	–	–
4.344 (0.614-30.709)	**self-reported basal body temperature**	–
0.775 (0.730-0.814)	0.442 (0.102-0.847)	**reference standard**

Specificity		
**wearable devices**	–	–
2.920 (1.302-6.552)	**self-reported basal body temperature**	–
0.886 (0.846-0.917)	0.727 (0.563-0.847)	**reference standard**

Positive likelihood ratio		
**reference standard**	–	–
1.472 (0.359-6.026)	**wearable devices**	–
5.693 (3.158-10.262)	3.868 (0.839-17.828)	**self-reported basal body temperature**

Negative likelihood ratio		
**reference standard**	–	–
0.680 (0.253-1.830)	**wearable devices**	–
0.250 (0.204-0.307)	0.368 (0.134-1.011)	**self-reported basal body temperature**

Diagnostic odds ratio		
**reference standard**	–	–
2.199 (0.199-24.293)	**wearable devices**	–
20.545 (6.550-64.439)	9.341 (0.653-133.556)	**self-reported basal body temperature**

Data presented the odds ratio (OR) comparing the sensitivity and specificity, and the ratio of positive likelihood ratio (PLR), negative likelihood ratio (NLR), and dagnostic odd ratio (DOR) of the detecting method in the column to that of the detecting method in the row.

Supplementary Table 4.

League table of the comparison of accuracy of wearable digital technology in detecting different fertility window intervals

**ovulation day**	-	-	-	-	-
-1.551 (-7.512, 4.445)	**± 1 day**	-	-	-	-
-3.056 (-9.295, 3.390)	-1.501 (-7.331, 4.518)	**± 2 days**	-	-	-
-3.541 (-9.561, 2.388)	-1.985 (-6.581, 2.496)	-0.487 (-6.588, 5.318)	**± 3 days**	-	-
34.154 (-42.473, 193.820)	35.730 (-40.838, 195.475)	37.205 (-39.344, 196.801)	37.769 (-38.820, 197.427)	**-5 days to ovulation**	-
-34.620 (-91.519, -12.075)	-33.021 (-89.914, -10.639)	-31.538 (-88.388, -9.273)	-30.974 (-87.688, -8.741)	-69.736 (-235.564, -9.088)	**reference standard**

Data presented the log-transformed odds ratio (log OR) comparing the accuracy of detecting method in the column to that of detecting method in the row or reference standard. A positive value indicates the method in the column showed higher accuracy than the method in the row in detecting fertility window, whereas a negative value indicates the method in the column is less accurate than the method in the row.

Supplementary Table 5.

League table of the comparison of the accuracy of the designs of wearable digital technology in detecting fertility window

**distal BT**	-	-	-	-	-	-	-	-	-
-0.009 (-0.370, 0.345)	**proximal BT**	-	-	-	-	-	-	-	-
0.002 (-0.130, 0.134)	0.008 (-0.358, 0.388)	**multiple parameters**	-	-	-	-	-	-	-
-0.001 (-0.186, 0.182)	0.005 (-0.313, 0.334)	-0.003 (-0.216, 0.204)	**single BT**	-	-	-	-	-	-
0.002 (-0.134, 0.134)	0.010 (-0.355, 0.383)	-0.000 (-0.139, 0.139)	0.002 (-0.206, 0.217)	**random forest model**	-	-	-	-	-
0.001 (-0.193, 0.198)	0.006 (-0.359, 0.398)	0.000 (-0.218, 0.216)	0.003 (-0.217, 0.219)	-0.000 (-0.221, 0.217)	**linear mixture model**	-	-	-	-
-0.008 (-0.315, 0.293)	0.000 (-0.316, 0.329)	-0.009 (-0.335, 0.303)	-0.005 (-0.277, 0.270)	-0.010 (-0.336, 0.306)	-0.010 (-0.336, 0.329)	**other AI model**	-	-	-
0.002 (-0.115, 0.117)	0.007 (-0.346, 0.370)	0.000 (-0.134, 0.134)	0.002 (-0.178, 0.193)	-0.001 (-0.135, 0.136)	-0.001 (-0.192, 0.197)	0.011 (-0.297, 0.321)	**band**	-	-
-0.014 (-0.525, 0.500)	-0.001 (-0.584, 0.568)	-0.015 (-0.538, 0.501)	-0.011 (-0.509, 0.496)	-0.014 (-0.545, 0.503)	-0.012 (-0.561, 0.519)	-0.006 (-0.516, 0.514)	-0.015 (-0.539, 0.494)	**ring**	-
-7.239 (-10.928, -6.075)	-7.236 (-10.865, -6.058)	-7.240 (-10.916, -6.073)	-7.236 (-10.884, -6.035)	-7.241 (-10.932, -6.056)	-7.243 (-10.948, -6.038)	-7.235 (-10.874, -5.995)	-7.238 (-10.921, -6.068)	-7.274 (-10.934, -5.902)	**reference standard**

Data presented the log-transformed odds ratio (log OR) comparing the accuracy of detecting method in the column to that of detecting method in the row or reference standard. A positive value indicates the method in the column showed higher accuracy than the method in the row in detecting fertility window, whereas a negative value indicates the method in the column is less accurate than the method in the row. Abbreviation: body temperature (BT), artificial intelligence (AI).

Supplementary table 6.

Interval of fertility window detection in wearable digital technology and other methods

Author	Type	Interval
Niggli A.,2023	WDT	±2 days
Yu J.L.,2022	WDT	-5 days to ovulation
Zhu T.Y.,2021	WDT	±2 days
Goodale B.M.,2019	WDT	-5 days to ovulation
B.S.H.,2022	WDT	±1 day, ±3 days
Shilaih M.,2018	WDT	ovulation day
Li Q.,2024	WDT	ovulation day, ±1 day, ±2 days, ±3 days
Gombert-Labedens M., 2024	WDT	-5 days to ovulation
Luo L.,2020	WDT	±3 days
Prasannan R.,2020	WDT	-5 days to ovulation
Regidor P.A.,2018	WDT	±3 days
Weiss G.,2022	WDT	±3 days
Sato D., 2024	WDT	NA
Zhu T.Y.,2021	self-reported BBT	±2 days
Luo L.,2020	self-reported BBT	±3 days
Martinez A.,1992	self-reported BBT	ovulation day, ±1 day, ±2 days
Tabbaa S., 2024	self-reported BBT	±3 days
Sato D., 2024	self-reported BBT	NA
Guermandi E.,2001	self-reported BBT	±3 days
Behre H.M.,2000	electronic hormone testing system	ovulation day
Pattnaik S.,2023	electronic hormone testing system	±3 days
Mu, Q.,2023	electronic hormone testing system	NA
Barron M. L.,2018	electronic hormone testing system	NA
Bouchard T. P.,2019	electronic hormone testing system	NA
Wegrzynowicz, A. K.,2022	electronic hormone testing system	NA
MacGregor E. A.,2005	electronic hormone testing system	NA
Thakur R. ,2020	electronic hormone testing system	±2 days
Mouriki. E,2019	calendar estimation	NA
Johnson S.,2018	calendar estimation	-5 days to ovulation
Suman S., 2023	calendar estimation	NA

Abbreviation: basal body temperature (BBT), wearable digital technology (WDT), Detection of exact ovulation day (ovulation day), Detection of one day before to after ovulation (± 1 day), Detection of 2 days before to after ovulation (± 2 days), Detection of 3 days before to after ovulation (± 3 days), Detection of 5 days in advance to ovulation until the ovulation day (-5 days to ovulation).

Supplementary Table 7. Abbreviation

Full name	Abbreviation
artificial intelligence	AI
basal body temperature	BBT
body mass index	BMI
body temperature	BT
Clearblue Fertility Monitor	CBFM
confidence interval	CI
diagnostic odds ratio	DOR
estradial	E2
estriol	E3G
finger skin temperature	FST
follicle stimulating hormone	FSH
Grading of Recommendations, Assessment, Development and Evaluations	GRADE
heart rate	HR
heart rate variability	HRV
linear mixed models	LMM
luteinizing hormone	LH
Markov chain Monte Carlo	MCMC
network meta-analysis	NMA
not appliable	NA
negative likelihood ratio	NLR
oestrone-3-glucuronide	E1G
polycystic ovary syndrome	PCOS
positive likelihood ratio	PLR
potential scale reduction factor	PSRF
pregnanediol-3-glucuronide	PdG
Quality Assessment of Diagnostic Accuracy Studies–Revised	QUADAS-2
random forest	RF
respiratory rate	RR
skin perfusion	SP
standard deviation	SD
summary receiver operating characteristic	SROC
surface under the cumulative ranking curve	SUCRA
wearable digital technology	WDT
wrist skin temperature	WST

PRISMA NMA Checklist

The Diagnostic Accuracy of Wearable Digital Technology to Detect Fertility Window and Menstrual Cycles: A Bayesian Network Meta-analysis

Section/Topic	Item #	Checklist Item	Reported on Page #
**TITLE**			
Title	1	Identify the report as a systematic review *incorporating a network meta-analysis (or related form of meta-analysis).*	1
**ABSTRACT**			
Structured summary	2	Provide a structured summary including, as applicable:<br>**Background:** main objectives<br>**Methods:** data sources; study eligibility criteria, participants, and interventions; study appraisal; and *synthesis methods, such as network meta-analysis.*<br>**Results:** number of studies and participants identified; summary estimates with corresponding confidence/credible intervals; *treatment rankings may also be discussed. Authors may choose to summarize pairwise comparisons against a chosen treatment included in their analyses for brevity.*<br>**Discussion/Conclusions:** limitations; conclusions and implications of findings.<br>**Other:** primary source of funding; systematic review registration number with registry name.	2
**INTRODUCTION**			
Rationale	3	Describe the rationale for the review in the context of what is already known*, including mention of why a network meta-analysis has been conducted.*	3-4
Objectives	4	Provide an explicit statement of questions being addressed, with reference to participants, interventions, comparisons, outcomes, and study design (PICOS).	5
**METHODS**			
Protocol and registration	5	Indicate whether a review protocol exists and if and where it can be accessed (e.g., Web address); and, if available, provide registration information, including registration number.	19
Eligibility criteria	6	Specify study characteristics (e.g., PICOS, length of follow-up) and report characteristics (e.g., years considered, language, publication status) used as criteria for eligibility, giving rationale. *Clearly describe eligible treatments included in the treatment network, and note whether any have been clustered or merged into the same node (with justification).*	18-19

Information sources	7	Describe all information sources (e.g., databases with dates of coverage, contact with study authors to identify additional studies) in the search and date last searched.	18
Search	8	Present full electronic search strategy for at least one database, including any limits used, such that it could be repeated.	Supplementary note - searching strategy
Study selection	9	State the process for selecting studies (i.e., screening, eligibility, included in systematic review, and, if applicable, included in the meta-analysis).	21
Data collection process	10	Describe method of data extraction from reports (e.g., piloted forms, independently, in duplicate) and any processes for obtaining and confirming data from investigators.	21
Data items	11	List and define all variables for which data were sought (e.g., PICOS, funding sources) and any assumptions and simplifications made.	19-20
**Geometry of the network**	**S1**	Describe methods used to explore the geometry of the treatment network under study and potential biases related to it. This should include how the evidence base has been graphically summarized for presentation, and what characteristics were compiled and used to describe the evidence base to readers.	22-23
Risk of bias within individual studies	12	Describe methods used for assessing risk of bias of individual studies (including specification of whether this was done at the study or outcome level), and how this information is to be used in any data synthesis.	21
Summary measures	13	State the principal summary measures (e.g., risk ratio, difference in means). *Also describe the use of additional summary measures assessed, such as treatment rankings and surface under the cumulative ranking curve (SUCRA) values, as well as modified approaches used to present summary findings from meta-analyses.*	20, 23
Planned methods of analysis	14	Describe the methods of handling data and combining results of studies for each network meta-analysis. This should include, but not be limited to:<br>• *Handling of multi-arm trials;*<br>• *Selection of variance structure;*<br>• *Selection of prior distributions in Bayesian analyses; and*<br>• *Assessment of model fit.*	22-23
**Assessment of Inconsistency**	**S2**	Describe the statistical methods used to evaluate the agreement of direct and indirect evidence in the treatment network(s) studied. Describe efforts taken to address its presence when found.	22
Risk of bias across studies	15	Specify any assessment of risk of bias that may affect the cumulative evidence (e.g., publication bias, selective reporting within studies).	22
Additional analyses	16	Describe methods of additional analyses if done, indicating which were pre-specified. This may include, but not be limited to, the following:<br>• Sensitivity or subgroup analyses;<br>• Meta-regression analyses;	22

		• *Alternative formulations of the treatment network; and*<br>• *Use of alternative prior distributions for Bayesian analyses (if applicable).*	
**RESULTS†**			
Study selection	17	Give numbers of studies screened, assessed for eligibility, and included in the review, with reasons for exclusions at each stage, ideally with a flow diagram.	6, Figure 1
**Presentation of network structure**	**S3**	Provide a network graph of the included studies to enable visualization of the geometry of the treatment network.	Figure 2 (b), Supplementary figure 5 (b)
**Summary of network geometry**	**S4**	Provide a brief overview of characteristics of the treatment network. This may include commentary on the abundance of trials and randomized patients for the different interventions and pairwise comparisons in the network, gaps of evidence in the treatment network, and potential biases reflected by the network structure.	6-7
Study characteristics	18	For each study, present characteristics for which data were extracted (e.g., study size, PICOS, follow-up period) and provide the citations.	6
Risk of bias within studies	19	Present data on risk of bias of each study and, if available, any outcome level assessment.	7
Results of individual studies	20	For all outcomes considered (benefits or harms), present, for each study: 1) simple summary data for each intervention group, and 2) effect estimates and confidence intervals. *Modified approaches may be needed to deal with information from larger networks.*	8-12
Synthesis of results	21	Present results of each meta-analysis done, including confidence/credible intervals. *In larger networks, authors may focus on comparisons versus a particular comparator (e.g. placebo or standard care), with full findings presented in an appendix. League tables and forest plots may be considered to summarize pairwise comparisons.* If additional summary measures were explored (such as treatment rankings), these should also be presented.	8-12
**Exploration for inconsistency**	**S5**	Describe results from investigations of inconsistency. This may include such information as measures of model fit to compare consistency and inconsistency models, *P* values from statistical tests, or summary of inconsistency estimates from different parts of the treatment network.	22
Risk of bias across studies	22	Present results of any assessment of risk of bias across studies for the evidence base being studied.	7
Results of additional analyses	23	Give results of additional analyses, if done (e.g., sensitivity or subgroup analyses, meta-regression analyses*, alternative network geometries studied,*	7, 9

		alternative choice of prior distributions for Bayesian analyses, and so forth).	
**DISCUSSION**			
Summary of evidence	24	Summarize the main findings, including the strength of evidence for each main outcome; consider their relevance to key groups (e.g., healthcare providers, users, and policy-makers).	13
Limitations	25	Discuss limitations at study and outcome level (e.g., risk of bias), and at review level (e.g., incomplete retrieval of identified research, reporting bias). *Comment on the validity of the assumptions, such as transitivity and consistency. Comment on any concerns regarding network geometry (e.g., avoidance of certain comparisons).*	15-16
Conclusions	26	Provide a general interpretation of the results in the context of other evidence, and implications for future research.	17
**FUNDING**			
Funding	27	Describe sources of funding for the systematic review and other support (e.g., supply of data); role of funders for the systematic review. This should also include information regarding whether funding has been received from manufacturers of treatments in the network and/or whether some of the authors are content experts with professional conflicts of interest that could affect use of treatments in the network.	24

PICOS = population, intervention, comparators, outcomes, study design.
* Text in italics indicateS wording specific to reporting of network meta-analyses that has been added to guidance from the PRISMA statement.
† Authors may wish to plan for use of appendices to present all relevant information in full detail for items in this section.
